# Low-Complexity Chase Decoding of Reed–Solomon Codes Using Channel Evaluation

**DOI:** 10.3390/e24030424

**Published:** 2022-03-18

**Authors:** Hao Wang, Wei Zhang, Yanyan Chang, Jiajing Gao, Yanyan Liu

**Affiliations:** 1 School of Microelectronics, Tianjin University, Tianjin 300072, China; ahao@tju.edu.cn (H.W.); tjuzhangwei@tju.edu.cn (W.Z.); yycsdut@tju.edu.cn (Y.C.); echo_gao0214@tju.edu.cn (J.G.); 2 College of Electronic Information and Optical Engineering, Nankai University, Tianjin 300071, China

**Keywords:** Reed–Solomon (RS) codes, low-complexity chase (LCC), algebraic soft-decision decoding (ASD), multiplicity assignment, low latency, VLSI

## Abstract

A novel time-varying channel adaptive low-complexity chase (LCC) algorithm with low redundancy is proposed, where only the necessary number of test vectors (TVs) are generated and key equations are calculated according to the channel evaluation to reduce the decoding complexity. The algorithm evaluates the error symbol numbers by counting the number of unreliable bits of the received code sequence and dynamically adjusts the decoding parameters, which can reduce a large number of redundant calculations in the decoding process. We provide a simplified multiplicity assignment (MA) scheme and its architecture. Moreover, a multi-functional block that can implement polynomial selection, Chien search and the Forney algorithm (PCF) is provided. On this basis, a high-efficiency LCC decoder with adaptive error-correcting capability is proposed. Compared with the state-of-the-art LCC (TV = 16) decoding, the number of TVs of our decoder was reduced by 50.4% without loss of the frame error rate (FER) performance. The hardware implementation results show that the proposed decoder achieved 81.6% reduced average latency and 150% increased throughput compared to the state-of-the-art LCC decoder.

## 1. Introduction

The error number of the codeword in the time-varying channel has great randomness, which makes it redundant to realize channel decoding with fixed decoding parameters. Therefore, channel evaluation plays an important role in channel decoding. It is of great significance to analyze the reliability of the channel in real-time by the received code sequence and automatically adjust the decoding parameters so that the channel decoder can maintain an efficient and low redundancy state on the premise of meeting the decoding performance.

Low-complexity chase (LCC) [1] is an excellent algebraic soft-decision decoding (ASD) algorithm for medium-to-high rate Reed–Solomon (RS) codes, which can achieve comparable error correction performance with other ASD algorithms, e.g., the Kötter–Vardy (KV) algorithm [2] and Bit-level Generalized Minimum Distance (BGMD) algorithm [3], while having lower complexity. As the main benefit of the LCC decoder, one level of multiplicity makes it possible to replace the interpolation and factorization stages by reformulated inversionless Berlekamp–Massey (RiBM) algorithm [4,5].

However, the LCC algorithm needs to judge the error symbol number of the codeword to dynamically adjust the decoding parameters for successfully decoding, otherwise it may cause a lot of redundant operations. The research work on the LCC algorithm mainly focuses on the selection of test vectors (TVs) and the design of high efficient decoders in recent years. A unified syndrome computation (USC)-based LCC decoder was proposed in [6]. The hardware of multiplicity assignment (MA) was implemented using the received bit-level magnitudes to evaluate the symbol reliability values [7].

However, the low hardware speed of this module limits the performance of the whole decoder. After that, an early termination algorithm for improving the throughput of the serial LCC decoder was proposed in [8]. In [9], a novel set of TVs derived from the analysis of the symbol error probabilities was applied to the modified LCC decoding. A LCC decoding algorithm using the module basis reduction (BR) interpolation technique [10], namely the LCC-BR algorithm, was proposed to reduce decoding complexity and latency. In addition, the number of 1 in the first syndrome $S_{0}$ can be used to infer whether the number of errors is even or odd, so as to reduce the number of TVs by half [11].

Thus far, there have been some preliminary studies on the decoding of time-varying channels. In [12], three different unreliable symbol position numbers η are used to realize channel environment adaptation; however, no specific channel evaluation scheme is given. An et al. [13] explored a classification decoding method based on deep learning to save decoding time. One of the most critical problems in the time-varying channel is decoding redundancy, which results in a large decoding latency. The decoding latency greatly limits the high-speed processing ability of the LCC decoder for massive data.

This study aimed to dynamically adjust the decoding parameters, reduce the decoding redundancy and improve the real-time communication performance by evaluating the number of errors in the received code sequence. The main contributions of the paper are summarized as follows.

(1) A time-varying channel adaptive LCC decoding algorithm is presented based on the channel soft information. It evaluates the time-varying channel environment and generates a suitable number of TVs and syndromes to reduce decoding redundancy and decoding delay.

(2) To improve the hardware performance of the multiplicity assignment (MA) block, we provide a simplified MA scheme and its hardware architecture. We also propose a new multi-functional block that can implement polynomial selection, Chien search and Forney algorithm (PCF) for saving hardware resources.

(3) A high-performance time-varying channel adaptive LCC decoder is provided. The decoding delay of the decoder is greatly reduced, and the hardware efficiency is significantly improved.

The rest of the paper is as follows. Section 2 introduces the conventional LCC decoding algorithm. The time-varying channel adaptive LCC algorithm is presented in Section 3. Section 4 introduces the simplified MA scheme, PCF block and the proposed LCC decoder. The implementation results are provided in Section 5. Section 6 draws the conclusion.

## 2. LCC Decoding Algorithm

The (n,k) RS codes over finite field $GF(2^{m})$ are modulated by binary phase-shift keying (BPSK) and transmitted over an additive white Gaussian noise (AWGN) channel, where *n* is the code symbol length, *k* is the message symbol length, *m* denotes the number of bits per symbol and $n=2^{m}-1$. The field elements are $GF\left(2^{m}\right)=\left\{0,1,\alpha ,\alpha^{2},\cdots ,\alpha^{n-1}\right\}$ with the primitive element α. The LCC decoder compares the reliability of each symbol $r_{i}$ in one codeword at the MA stage. The reliability of the *i*-th received symbol is defined by first hard-decision value $y_{i}^{HD}$ and the second hard-decision value $y_{i}^{2HD}$ as

(1) $$\Gamma_{i}=\mathrm{lg}\left[p\left(r_{i}|y_{i}^{HD}\right)/p\left(r_{i}|y_{i}^{2HD}\right)\right].$$

The smaller $\Gamma_{i}$ is, the less reliable the symbol is. The hard-decision $y_{i}^{HD}$ and $y_{i}^{2HD}$ can be obtained by flipping the bit with the lowest reliability (i.e., the minimum level value). η unreliable symbols with the smallest values of $\Gamma_{i}$ are selected to generate multiple TVs that are most likely to achieve successful decoding. $2^{\eta }$ TVs can be obtained by combining $y_{i}^{HD}$ or $y_{i}^{2HD}$ of the η unreliable symbols. If there exists a TV whose error symbol number is less than the decoding radius *t* in the TV set Γ (TV1,TV2,⋯, TV$2^{\eta }$), where $t=\frac{n-k}{2}$, the decoding can succeed.

## 3. Time-Varying Channel Adaptive LCC Algorithm

**Figure 1:**
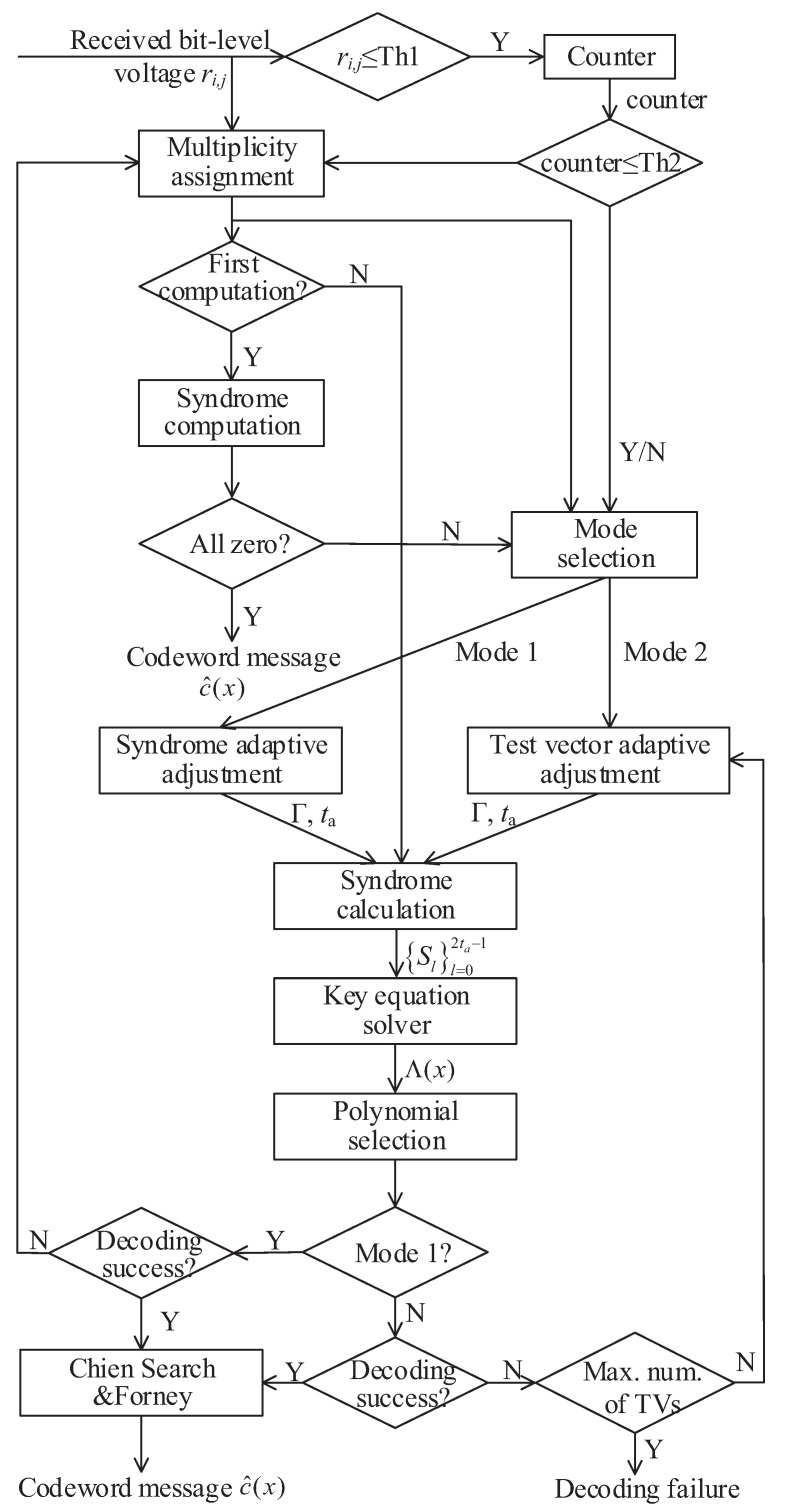
Data flow of time-varying channel adaptive LCC decoding.

In the time-varying channel, the signal-to-noise ratio (SNR) changes rapidly with time, which makes the LCC decoding algorithm with fixed parameters cause a great deal of decoding redundancy and delay. To solve this problem, we propose a novel LCC decoding algorithm in Algorithm 1 to reduce the number of TVs and syndromes, and the flowchart is shown in Figure 1. The main idea of the algorithm is to evaluate the error symbol number of each codeword by counting the number of unreliable bits in order to dynamically adjust the number of TVs and the number of syndromes used to achieve successful decoding.

In step 1, the error symbol number and unreliable value of the codeword are evaluated by the received bit-level value $r_{i,j}$, where *i* is the symbol position and *j* is the bit position. The number of bits in each codeword whose bit-level value is less than the threshold Th1 (set to 0.3 in this paper) is counted. For each symbol, the smallest bit $\gamma_{i}$ is selected, and the unreliable bit position set Δ is updated.

In step 2, two modes are set, one is the syndrome adaptive adjustment, and the other is the TV adaptive adjustment. First, if the syndromes calculated from the first test vector TV1 are all zero, then the codeword is correct. Otherwise, if the unreliable bit number (i.e., counter) is less than Th2, mode 1 is performed. The threshold Th2 used to distinguish e≤t and *e* > *t* is the number of bits whose level values are less than Th1, where *e* is the number of error symbols.

If counter ≤ Th2, which indicates that the number of error symbols does not exceed *t*, hard decision decoding (HDD) can be adopted, otherwise, the LCC decoding can be selected. On the premise of ensuring decoding performance, Th2 can be taken as data equal to 2t. For the standard (255,239) RS codes, Th2 values can be 16 as shown in Table 1. If some performance is lost in exchange for decoding speed, the value of Th2 can be appropriately increased. The syndrome *S*, error location β and error value δ satisfy the following equations:

(2) $$\begin{array}{c} S_{0}=\delta_{1}\beta_{1}+\delta_{2}\beta_{2}+\delta_{3}\beta_{3}+\cdots +\delta_{e}\beta_{e}\\ S_{1}=\delta_{1}\beta_{1}^{2}+\delta_{2}\beta_{2}^{2}+\delta_{3}\beta_{3}^{2}+\cdots +\delta_{e}\beta_{e}^{2}\\ \vdots \\ S_{2t-1}=\delta_{1}\beta_{1}^{2t}+\delta_{2}\beta_{2}^{2t}+\delta_{3}\beta_{3}^{2t}+\cdots +\delta_{e}\beta_{e}^{2t}. \end{array}$$

When e<t, only the first 2e syndromes need to be calculated to get the error vector. The number of equations to be solved is reduced from 2t to 2e, which can reduce the redundant calculation of 2t−2e equations. If counter > Th2, we should make full use of the channel soft information to achieve a larger decoding radius. The selected number of TVs can be determined by the counter, which is obtained by statistical regulation. Table 1 shows the corresponding relationship between the counter value of the received (255,239) RS code and the maximum possible number of error symbols and gives the corresponding values of $t_{a}$ and the number of TVs. Table 2 shows the parameter correspondence of (127,119) RS code.
**Algorithm 1:** Time-Varying Channel Adaptive LCC Algorithm
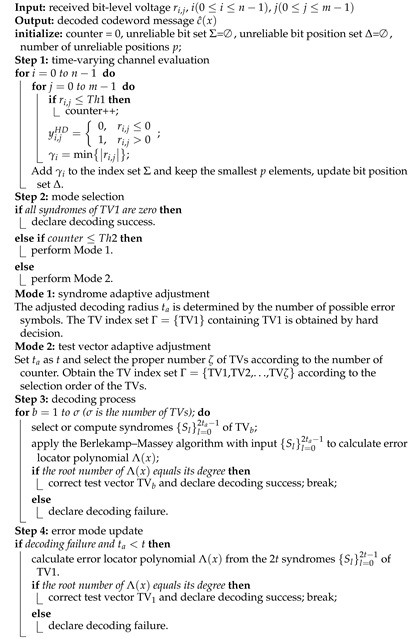


In addition, in order to find the TV that can be successfully decoded with fewer attempts, we introduce two TV selection and sorting methods. After determining the TV1, we need to find the TV2 based on the idea of decoding complementarity, then find the TV3 complementary to their decoding based on the previous two TVs and constantly repeat the above rules to obtain the TV index set Γ={TV1,TV2,…,TVζ}. It can be concluded from [11] that when the signal to noise ratio (SNR) exceeds 6.1 dB, the probability that the symbol error has a 1-bit error is above 99%.

The first syndrome is $S_{0}=R\left(\alpha^{0}\right)=\sum_{l=1}^{e}E_{l}$, where R(x) represents the received codeword and $E_{l}$ is the error vector. According to the parity number of 1 contained in $S_{0}$ and the idea of compensation decoding, two sets of index sets, odd index set $\Gamma_{o}=\{$TV${}_{o}1$,TV${}_{o}2$,…,TV${}_{o}\zeta \}$ and even index set $\Gamma_{e}=\{$TV${}_{e}1$,TV${}_{e}2$,…,TV${}_{e}\zeta \}$, are tested. Table 3 shows the sequence of 16 TVs of decoding (255,239) RS codes obtained through simulation, and each TV is the best compensation for all previous TVs. The order of eight TVs for decoding (127,119) RS codes is given in Table 4. In addition, the corresponding optimal test order when the number of 1 in $S_{0}$ is odd or even is given.

In step 3, the (n,n−2t) RS code is regarded as $\left(n,n-2t_{a}\right)$ RS code, and the first 2$t_{a}$ syndromes are used to complete decoding. The number of iterations for solving the key equation changed from 2t times to $2t_{a}$ times, reducing $2(t-t_{a})$ iterations. Most of the corresponding relations are reliable; however, there are a few counter values that can not accurately reflect the error symbol number of codewords. Therefore, we introduce the error mode update method in step 4 to reduce the impact of the above problems on the decoding performance. The decoding failed codewords in the syndrome adaptive adjustment are corrected by the 2t syndromes ${\left\{S_{l}\right\}}_{l=0}^{2t-1}$ of TV1 again.

**Figure 2:**
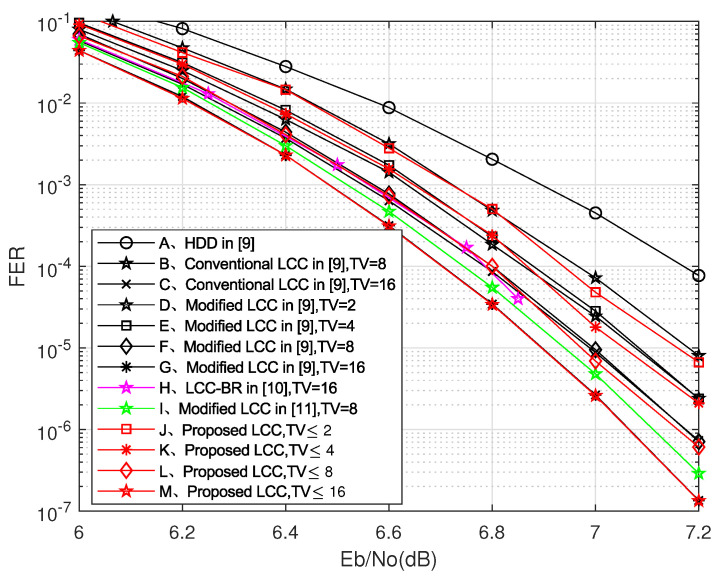
Frame error rate (FER) versus Eb/No of the proposed decoder compared with other algorithms for RS(255,239) codes.

The simulation results of the proposed LCC algorithm for (255,239) RS code are given and compared with several previous algorithms in Figure 2. The proposed LCC algorithm reduces the decoding complexity by dynamically adjusting the number of TVs. Compared with the current state-of-the-art LCC decoding algorithms [9,10,11], the proposed LCC decoding algorithm has achieved equivalent or better decoding performance with fewer TVs. The amount of computation of our algorithm is greatly reduced. We simulate the time-varying channel, which randomly changes with frame in the range of [6.5, 8] dB and simulate the decoding performance of 800,000 codewords through different algorithms.

We give the total number and average number of TVs required to decode these codewords. As shown in Table 5, compared with the cases of TV = 8 and TV = 16 in [9], the proposed LCC decoding algorithm achieves better decoding performance while reducing TVs by 23.5% and 50.4%, respectively. Compared with the case of TV = 8 in [11], the proposed LCC decoding algorithm achieves a performance gain of 43.1% when the number of TVs is approximately equal.

**Figure 3:**
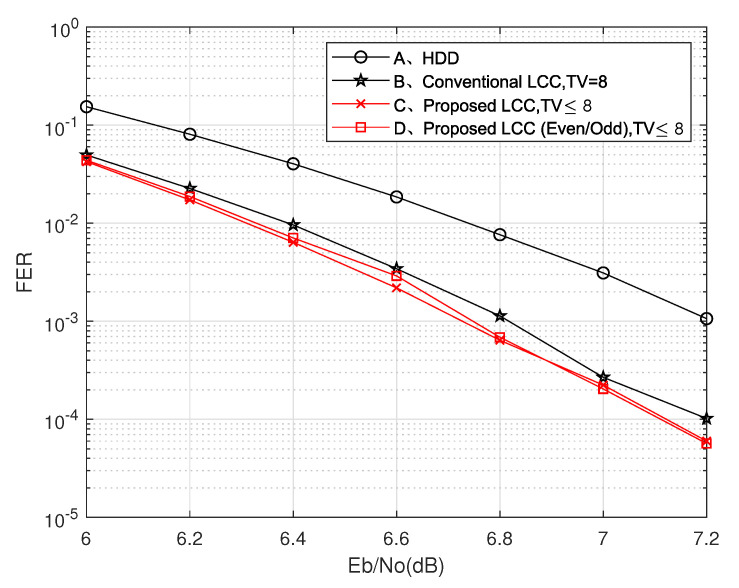
Frame error rate (FER) versus Eb/No of the proposed decoder compared with other algorithms for RS(127,119) codes.

Figure 3 shows the decoding performance of the proposed decoder with other algorithms for (127,119) RS codes. The proposed scheme obtains better performance than the traditional LCC algorithm with fewer TVs. When the channel conditions are poor, the performance of the LCC decoding scheme based on a single test set is better because the probability of multi-bit errors in the symbol is greater when the channel conditions are poor, which makes the error parity judgment confused. However, when the channel conditions are good, the performance of the LCC decoding scheme based on the parity test set is better. Comparisons in the time-varying channel of [6.5, 8] dB are also provided. As shown in Table 6, the proposed LCC decoding algorithm reduces the number of TVs by 52.4%, which also achieves more competitive decoding performance than the traditional LCC decoding algorithm.

## 4. The Proposed Time-Varying Channel Adaptive LCC Decoder

**Figure 4:**
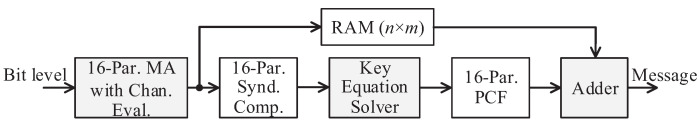
Block diagram for the time-varying channel adaptive LCC decoder.

This section presents the architecture of the proposed time-varying channel adaptive LCC decoder, which processes up to 16 test vectors in the set. As shown in Figure 4, the proposed decoder consists of four blocks: 16-parallel MA with channel evaluation, 16-parallel syndrome computation (SC), key equation solver (KES), 16-parallel PCF block. An n×m size RAM is used to cache the codeword.

**Figure 5:**
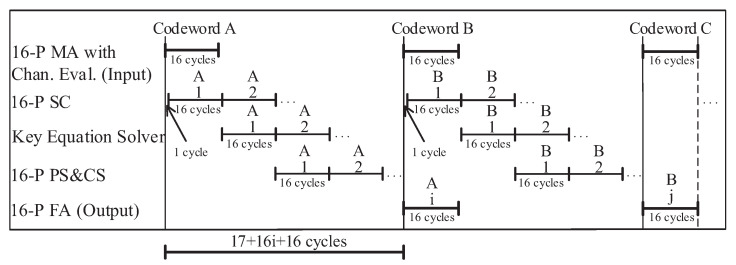
The timing diagram of the proposed LCC decoder.

The decoding chronogram of the proposed decoder is shown in Figure 5. As the timing diagram shows, when the 16-parallel MA block generates the TVs, the 16-parallel SC block only delays one clock cycle to start working, and both blocks need 16 clock cycles.

Thus, it only takes 17 clock cycles from the input of the first codeword A to the output of the first TV’s syndromes. It takes 16 clock cycles for the KES block to compute the error locator polynomial Λ(x) and the error magnitude polynomial Ω(x) through the RiBM algorithm. At the same time, the 16-parallel SC block calculates the syndromes of the second TV. Then, the 16-parallel PCF block completes PS and Chein search (CS) after 16 clock cycles. If the order of the error location polynomial is lower than *t*, the decoder will complete the calculation of Forney algorithm by reusing the PCF block, which takes 16 clock cycles.

Otherwise, it will continue to judge the next TV. Since the first TV is the best hard decision value of the received codeword, it can be successfully decoded in most cases. If all the syndromes are zero, the TV will be output directly. If $t_{a}<t$, the KES block only needs 2$t_{a}$ clock cycles to calculate the equation; however, other blocks still need 16 clock cycles to complete their functions.

The latency of the proposed LCC decoder is 17 + 16 × *i* + 16 cycles. When *i* (i.e., the ordinal number of the TV) takes the maximum value of 16, the maximum latency of the proposed decoder is 17 + 16 × 16 + 16 = 289 cycles. When the minimum value of *i* is 1, the minimum latency of the proposed decoder is 17 + 16 × 1 + 16 = 49 cycles.

### 4.1. Multiplicity Assignment with Channel Detection

The existing MA scheme [7] needs to realize $\log_{2}m$ times pairwise comparisons through the comparator to select the most unreliable bit of each symbol, which greatly limits the hardware performance. To solve this issue, we propose a simplified MA scheme by making full use of the channel soft information. If the level value $r_{i,j}$ is less than Th1, it is considered as the most unreliable bit $\gamma_{i}$ of this symbol.

(3) $$\gamma_{i}=r_{i,j}$$

Otherwise, the first bit $r_{i,0}$ in the symbol is considered unreliable.

(4) $$\gamma_{i}=r_{i,0}$$

Statistics show that the probability of two or more unreliable bits in a symbol is very small. If multiple unreliable bits appear in a symbol, the last bit less than Th1 is selected as the unreliable bit. Each update of the MA block keeps only the first and second decision values of the *p* most unreliable elements and their location information.

**Figure 6:**
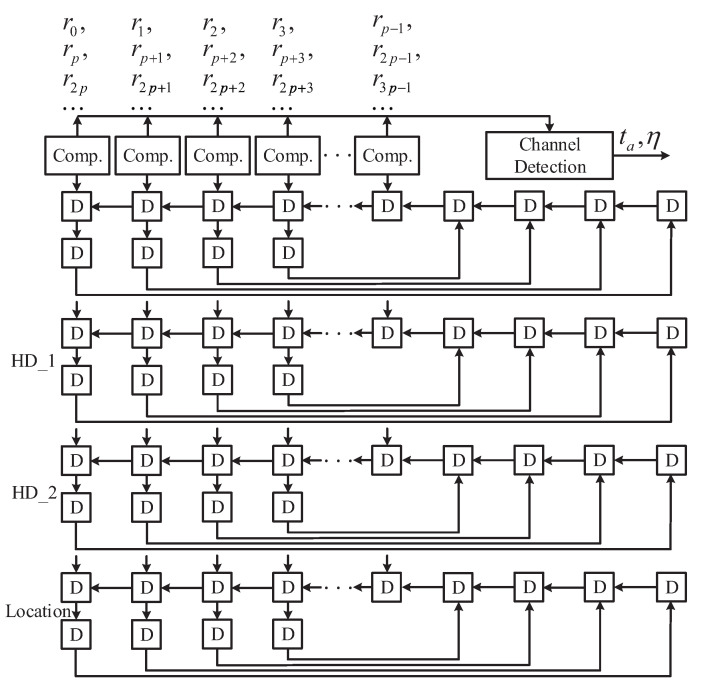
The architecture of the proposed MA.

The simulation results in Table 7 show that the proposed MA block can achieve the maximum clock frequency of 385 MHz in SMIC 0.13 μm CMOS technology, whose throughput is 75% higher than the MA in [7]. In addition, the hardware area and power consumption are also reduced. A *p*-degree parallel MA architecture including *p* (p=16) comparators is shown in Figure 6, which only needs ⌈255/p⌉ clock cycles to complete MA.

### 4.2. The Architecture of the Proposed Pcf Block

**Figure 7:**
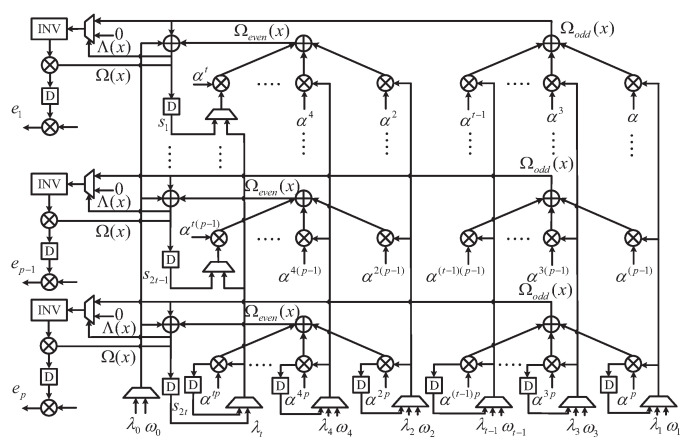
The architecture of the proposed PCF.

The proposed *p*-parallel PCF block shown in Figure 7 is an upgraded module of [14], which only needs 16 clock cycles to realize PS and Chein search at the same time. If the polynomial is selected correctly, the block can reuse the basic unit to complete the *p*-way (p=16) Forney algorithm.

## 5. Implementation Results

The proposed time-varying channel adaptive LCC decoder of (255,239) RS code was implemented using 0.13 μm and 65 nm CMOS process. As shown in Table 8, the maximum clock frequency of the proposed decoder is 385 MHz in 0.13 μm process and 550 MHz in 65 nm process by Synopsys design tools. Compared with the current state-of-the-art LCC decoders [9,11], the proposed LCC decoder achieves equivalent or better coding gain. However, the high clock frequency and additional hardware resources also increase the power consumption of the proposed decoder.

For the time-varying channel with the range of [6.5, 8] dB, the proposed decoder requires an average of 1.06 TVs to complete decoding. The average latency of the decoder is 17 + 1.06 × 16 + 16 = 50 cycles, which is 81.6% smaller than that of the LCC decoder in [11]. The average throughput of the proposed decoder can be calculated by Formula (5).

(5) $$\;\mathrm{Throughput}\;=\frac{\#\;\mathrm{of}\;\mathrm{bits}\;\mathrm{processed}\;\mathrm{each}\;\mathrm{time}\;}{\#\;\mathrm{of}\;\mathrm{clock}\;\mathrm{cycles}\;\mathrm{needed}\;\times \;T_{\min\;}}$$

The estimated throughput is (255 × 8 × 385)/50 = 15.7 Gb/s in 0.13 μm process. The estimated throughput is (255 × 8 × 550)/50 = 22.4 Gb/s in the 65 nm process.

Compared with [7,9,11], the throughput (5) of the proposed decoder is increased by 8.8, 4.1 and 1.5 times, respectively. Compared with the state-of-the-art LCC decoder, the proposed decoder has lower latency and higher throughput, which indicates that the proposed decoder has better hardware performance.

## 6. Conclusions

In this paper, we derived a time-varying channel adaptive LCC algorithm, which can dynamically adjust the decoding parameters through channel detection. The proposed algorithm can greatly reduce the redundant TVs and key equations, thus, reducing the decoding complexity and latency. To evaluate the algorithmic performance, we performed effect analysis in the time-varying channel.

Compared to the state-of-the-art LCC algorithm, the number of TVs required was reduced by 50.4%, and the average latency of the decoder was reduced by 81.6%. To reduce the pairwise comparison times of selecting the most unreliable bit for each symbol in the MA block, a simplified scheme assisted by setting a bit-level threshold was proposed to improve its hardware efficiency. In addition, a PCF block was proposed to reduce the consumption of hardware resources.

Based on the above techniques, the hardware architecture of the proposed time-varying channel LCC decoder is presented. Compared to previously published papers, the implementation results show that the proposed LCC decoder achieved at least 150% throughput improvement. Future work will focus on high-performance time-varying LCC decoders.

## Figures and Tables

**Table 1:** The corresponding relationship between the counter value of received RS(255,239) code and the decoding parameters.

Counter	2	4	6	8	10	12	14	16	24	36	>36
Error num.	1	2	3	4	5	6	7	8	9	10	>10
$t_{a}$	1	2	3	4	5	6	7	8	8	8	8
TV num. ζ	1	1	1	1	1	1	1	1	4	8	16

**Table 2:** The corresponding relationship between the counter value of received RS(127,119) code and the decoding parameters.

Counter	2	4	6	8	14	>14
Error num.	1	2	3	4	5	>5
$t_{a}$	1	2	3	4	4	4
TV num. ζ	1	1	1	1	4	8

**Table 3:** The selection order of test vectors for RS(255,239) codes.

Testing Order	TV1	TV2	TV3	TV4
Flipping pattern	00000000	11111000	10000110	11100100
Testing order	TV5	TV6	TV7	TV8
Flipping pattern	01011011	10100011	01110000	00111010
Testing order	TV9	TV10	TV11	TV12
Flipping pattern	11001001	00010101	10000000	01001110
Testing order	TV13	TV14	TV15	TV16
Flipping pattern	00101001	11010010	01000101	10011100

**Table 4:** The selection order of test vectors for RS(127,119) codes.

Testing Order	TV1	TV2	TV3	TV4
Flipping pattern	00000000	11100000	10011000	01000110
Testing order	TV5	TV6	TV7	TV8
Flipping pattern	00110000	00101001	11000101	10000010
Testing order	TV${}_{e}$1	TV${}_{e}$2	TV${}_{e}$3	TV${}_{e}$4
Even Flipping pattern	00000000	11100000	10011000	01010100
Testing order	TV${}_{e}$5	TV${}_{e}$6	TV${}_{e}$7	TV${}_{e}$8
Even Flipping pattern	00100011	10101100	01001000	10000011
Testing order	TV${}_{o}$1	TV${}_{o}$2	TV${}_{o}$3	TV${}_{o}$4
Odd Flipping pattern	00000000	11100000	10011000	00000100
Testing order	TV${}_{o}$5	TV${}_{o}$6	TV${}_{o}$7	TV${}_{o}$8
Odd Flipping pattern	01110010	11000101	10101010	00110001

**Table 5:** Comparison of the decoding performance for RS(255,239) codes.

Algorithm	Num. of TVs	Avg. Num. of TVs	FER
Conv. LCC in [9], TV = 8	6400 k	8	3.17 × 10${}^{-4}$
Conv. LCC in [9], TV = 16	12,800 k	16	1.66 × 10${}^{-4}$
Modified LCC in [9], TV = 8	6400 k	8	1.95 × 10${}^{-4}$
Modified LCC in [9], TV = 16	12,800 k	16	7.96 × 10${}^{-5}$
Modified LCC in [11], TV = 8	6400 k	8	1.34 × 10${}^{-4}$
Proposed LCC TV ≤ 8	4899 k	6	1.74 × 10${}^{-4}$
Proposed LCC TV ≤ 16	6347 k	8	7.63 × 10${}^{-5}$

**Table 6:** Comparison of the decoding performance for RS(127,119) codes.

Algorithm	Conv. LCC, TV = 8	Proposed LCC, TV ≤ 8	Proposed LCC (Even/Odd), TV ≤ 8
Num. of TVs	6400 k	3049 k	3049 k
Avg. num. of TVs	8	3.8	3.8
FER	7.88 × 10${}^{-4}$	6.71 × 10${}^{-4}$	6.76 × 10${}^{-4}$

**Table 7:** The implementation results of the proposed MA module in 0.13 μm CMOS process at 200 MHz.

Module	Area (${\mathrm{mm}}^{2}$)	Gate Count (XORs)	$f_{\max}$ (MHz)	Throughput (Gb/s)	Power (mW)
MA [7]	0.0661	5561	220	1.76	6.0
Proposed	0.0625	5261	385	3.08	5.6

**Table 8:** Implementation results of the LCC decoders.

Architecture	[7]	[9]	[11]	Proposed	Proposed
Tech.	0.13 µm	65 nm	65 nm	0.13 µm	65 nm
$f_{\max}$ (MHz)	220	550	550	385	550
Power (mW@MHz)	-	23.0@550	21.5@550	259.2@385	31.5@550
Throughput (Gbps)	1.6a	4.4a	8.8a	15.7b	22.4b
Gate count (kXORs) with buffer	27.9	31.1	34.9	55.4	56.8
Coding gain (dBs@FER)	0.37@10${}^{-6}$	0.56@10${}^{-6}$	0.50@10${}^{-6}$	0.56@10${}^{-6}$	0.56@10${}^{-6}$
Latency (clock cycles)	400a	528a	272a	50b	50b

a Fixed value b Average value.

## Data Availability

Not applicable.
